# Controlling long-range genomic interactions to reprogram the β-globin locus

**DOI:** 10.1186/1756-8935-6-S1-O39

**Published:** 2013-03-18

**Authors:** Wulan Deng, Jeremy W Rupon, Hongxin Wang, Andreas Reik, Philip D Gregory, Gerd A Blobel

**Affiliations:** 1 The Children’s Hospital of Philadelphia, Hematology, Philadelphia, PA, 19104, USA; 2 University of Pennsylvania, Department of Biology, Philadelphia, PA, 19104, USA; 3 Sangamo BioSciences, Inc, Richmond, CA, 94804, USA

## 

Distal enhancers physically contact target promoters to confer high level transcription. At the mammalian β-globin loci long-range chromosomal interactions between a distal enhancer, called the locus control region (LCR), and the globin genes are developmentally dynamic such that the LCR loops to the embryonic, fetal and adult globin genes in a stage-appropriate fashion. LCR-globin gene interactions require the nuclear factor Ldb1. Recently, we employed artificial zinc finger (ZF) proteins to target Ldb1 to the endogenous β-globin locus to force an LCR-promoter loop. This led to substantial activation of β-globin transcription and suggested that forced chromatin looping could be employed as a powerful tool to manipulate gene expression *in vivo* (Deng *et al*., *Cell* 2012). Reactivation of the fetal globin genes in adult erythroid cells has been a long-standing goal in the treatment of patients with sickle cell anemia. Therefore, building on our findings, we investigated whether the developmentally silenced embryonic globin gene βh1 can be re-activated in adult murine erythroblasts by re-directing the LCR away from the adult type globin gene and towards its embryonic counterpart. To this end, Ldb1 was fused to artificial ZF proteins (ZF-Ldb1) designed to bind to the βh1 promoter. ZF-Ldb1 was introduced into definitive erythroid cells in which only the adult but not the embryonic β-like globin gene is expressed. *In vivo* binding of ZF-Ldb1 to its intended target was verified by chromatin immunoprecipitation assay. Strikingly, expression of ZF-Ldb1 re-activated βh1 transcription up to approximately ~24% of total cellular β-globin production. This suggests that forced tethering of a looping factor to a select promoter can be employed to override a pre-existing developmental long-range chromatin interaction to reprogram a developmentally controlled gene locus.

